# Liver Enzyme Elevations in Rheumatoid Arthritis: Clinical Relevance and Influence on Treatment Strategies

**DOI:** 10.3390/jcm14207213

**Published:** 2025-10-13

**Authors:** Yahya Atayan, Servet Yolbas, Emin Bodakci

**Affiliations:** 1 Department of Gastroenterology, Medical Faculty, Inonu University, Battalgazi 44000, Türkiye; 2 Department of Rheumatology, Medical Faculty, Inonu University, Battalgazi 44000, Türkiye; servetyolbas@yahoo.com.tr; 3 Department of Gastroenterology, Gaziantep City Hospital, Gaziantep 27470, Türkiye; doktor.emin.0903@hotmail.com

**Keywords:** rheumatoid arthritis, methotrexate, hepatotoxicity

## Abstract

**Background and Aim**: Rheumatoid arthritis (RA) is a chronic inflammatory polyarthritis of unknown etiology that symmetrically involves the synovial joints and leads to erosive arthritis. However, when inflammation remains uncontrolled, it not only affects the joints but also increases the risk of various systemic complications, particularly cardiovascular diseases, osteoporosis, and malignancies such as lymphoma. Early initiation of disease-modifying antirheumatic drugs (DMARDs) has been shown to yield superior outcomes in terms of both clinical response and the prevention of joint damage. Nevertheless, the development of hepatotoxicity during treatment may necessitate dose adjustments or even modifications of the therapeutic protocol. Our aim in this study was to retrospectively evaluate the changes in liver enzyme levels in RA patients before and during treatment, especially in MTX and combination therapies using MTX, and to evaluate how these abnormalities affect treatment strategies. **Materials and Methods/Results**: Among the 33 patients included in this study, 15 exhibited elevated liver enzymes prior to treatment, whereas 18 developed hepatic enzyme abnormalities during therapy. Of the 12 patients receiving methotrexate (MTX) monotherapy and the 15 patients using MTX within a combination regimen, a total of 7 patients (21%) continued to present with elevated liver enzymes during follow-up. Among these, 5 patients (19%) were managed successfully by reducing the MTX dose, while MTX therapy had to be completely discontinued in 2 patients (7%). Notably, all 7 patients who required treatment modification due to persistent enzyme elevation belonged to the group with pre-existing liver enzyme abnormalities and were receiving MTX as part of a combination therapy regimen. **Conclusions**: These findings indicate that hepatotoxicity risk in RA patients can be effectively managed through close laboratory monitoring and timely dose reduction, with treatment discontinuation being required only in rare cases.

## 1. Introduction

Rheumatoid arthritis (RA) is a chronic inflammatory polyarthritis of unknown etiology that symmetrically involves the synovium and leads to erosive arthritis. The disease causes severe functional impairment due to cartilage and bone erosion–related joint destruction, and, if left untreated, significantly restricts daily life activities. Furthermore, uncontrolled inflammation not only affects the joints but also increases the risk of systemic complications such as cardiovascular diseases, osteoporosis, and malignancies. Therefore, early diagnosis and effective treatment strategies are of critical importance in the management of RA.

It has been well established in the literature that early initiation of disease-modifying antirheumatic drugs (DMARDs) results in superior outcomes, both in terms of clinical response and prevention of joint damage [1]. Contemporary meta-analyses and observational studies have demonstrated that methotrexate (MTX) provides efficacy comparable to, or even greater than, other available agents, including biologic therapies [2]. Nevertheless, despite its long-term effectiveness, MTX poses limitations for clinicians due to adverse effects, particularly hepatotoxicity. Clinical observations indicate that elevations in serum liver enzymes (LEs) occur in 15–50% of patients during long-term low- to moderate-dose MTX therapy, with approximately 5% experiencing elevations exceeding twice the upper limit of normal, thereby necessitating treatment discontinuation or dose adjustment [3,4]. However, how such enzyme abnormalities should be managed clinically, as well as the independent contribution of MTX to hepatotoxicity, remains controversial [5].

Existing studies in the literature generally address MTX-associated LE elevations from a single perspective, focusing either solely on changes during treatment or on the incidence of hepatotoxicity. Yet, studies evaluating both pre-treatment elevations and treatment-related LE changes within the same patient population are extremely limited. The aim of this study, therefore, is to retrospectively assess liver enzyme alterations before and during treatment in RA patients and to analyze how these abnormalities influence treatment decisions. In doing so, the study not only reports the frequency of hepatotoxicity but also provides practical insights into the clinical management of LE elevations. Thus, compared to the existing literature, it offers a more comprehensive perspective.

## 2. Materials and Methods

In this study, the medical records of 670 patients diagnosed with rheumatoid arthritis (RA), who were under regular follow-up and treatment at the Rheumatology Clinic of Inönü University Faculty of Medicine between August 2021 and August 2025, were retrospectively reviewed. Exclusion criteria were as follows: patients with elevated liver enzymes (LEs) and concomitant obesity (body mass index > 30 kg/m^2^) (*n* = 32); those with grade 2–3 fatty liver disease identified on whole-abdominal ultrasonography (*n* = 120); patients with active alcohol consumption (*n* = 15); patients with diabetes mellitus receiving treatment and follow-up (*n* = 43); patients positive for HBsAg, including all chronic hepatitis B cases regardless of whether they were treated (*n* = 18); patients positive for anti-HCV, including all chronic hepatitis C cases regardless of treatment status (*n* = 2); and patients without confounding comorbidities or liver enzyme elevations (*n* = 450). After applying these exclusion criteria, 33 patients were included in the final analysis. The study endpoint was defined as discontinuation of methotrexate (MTX) therapy in cases where patients with initially normal or mildly elevated liver enzymes developed alanine aminotransferase (ALT) and aspartate aminotransferase (AST) elevations exceeding three times the upper limit of normal (ULN) during RA treatment. Among the included patients, 19 were female and 14 were male, with a mean age of 66 ± 2 years. Fifteen patients had elevated liver enzymes before treatment, whereas 18 developed liver enzyme abnormalities during therapy. Regarding treatment regimens, 12 patients received MTX monotherapy, 4 received non-MTX monotherapy (hydroxychloroquine, leflunomide, etc.), 8 received MTX in combination with other DMARDs, 2 received biologic monotherapy (anti-TNF or rituximab), and 7 received a biologic agent combined with MTX. All patients initiated on MTX were treated according to Swedish guidelines, receiving oral MTX at a weekly dose of 7.5 mg, in combination with folic acid supplementation (at least 5 mg per week, administered on days other than MTX intake). The mean disease duration from RA diagnosis was 4 ± 2 years. Biochemical evaluation defined ALT and AST values exceeding the ULN as “elevated.” The mean treatment duration among patients who developed liver enzyme abnormalities was 3 ± 1 years. Follow-up laboratory assessments were performed initially at weeks 4–6 after treatment initiation, and subsequently at intervals of 8–12 weeks. Patients were categorized into three groups based on the degree of enzyme elevation: Grup 1: ALT and AST >1–2 × ULN, Grup 2: ALT and AST > 2–3 × ULN, Grup 3: ALT and AST > 3 × ULN. For comparative analyses between patients with and without LE elevations, all eligible cases were included to maximize statistical power. Intergroup comparisons were performed using the independent-samples *t*-test or Mann–Whitney U test for continuous variables, and chi-square or Fisher’s exact test for categorical variables. Logistic regression analyses (both univariate and multivariate) were applied to identify predictors of elevated liver enzymes. Statistical significance was set at *p* < 0.05.

## 3. Results

Among the 33 patients included in the study, 19 were female and 14 were male, with a mean age of 68 years in women and 64 years in men (Table 1). Over a mean follow-up period of three years, when classified according to liver enzyme (LE) elevations, 22 patients (66%) were categorized as Group 1, 7 patients (21%) as Group 2, and 4 patients (12%) as Group 3 (Table 2). No statistically significant difference was observed in the distribution of enzyme elevations by sex (Table 1). Analysis of treatment subgroups revealed that among the 12 patients receiving methotrexate (MTX) monotherapy, 11 (91%) were in Group 1 and only 1 patient (9%) in Group 2. Of the 8 patients treated with MTX in combination with other DMARDs, 6 (75%) were in Group 1 and 2 (25%) in Group 2. In the biologic monotherapy group (*n* = 2), 1 patient (50%) was classified in Group 1 and the other (50%) in Group 2. Among the 7 patients who received biologic agents combined with MTX, 1 (16%) was in Group 1, 3 (42%) in Group 2, and 3 (42%) in Group 3 (Table 3). When the subgroup of 15 patients with pre-treatment LE elevations was analyzed separately, 8 patients (53%) had elevations of 1–2 × ULN, 6 patients (40%) had elevations of 2–3 × ULN, and 1 patient (7%) exhibited elevations greater than 3 × ULN. Within this subgroup, 5 patients were treated with MTX monotherapy, 2 with non-MTX monotherapy, 3 with combination DMARD therapy, 2 with biologic monotherapy, and 3 with biologic plus DMARD combination therapy (Table 4). In terms of treatment-related changes, no statistically significant differences were observed regarding age or sex. Among the 12 patients receiving MTX monotherapy and the 15 patients using MTX within combination regimens, persistent LE elevations were noted in a total of 7 patients (21%). Of these, 5 patients (19%) were managed by dose reduction in MTX, while in 2 patients (7%), MTX therapy was completely discontinued. Importantly, all 7 patients requiring treatment modification due to LE elevations belonged to the subgroup that had pre-existing enzyme abnormalities prior to treatment initiation and were receiving MTX as part of a combination regimen. By contrast, no treatment discontinuations were required among patients receiving alternative therapies (non-MTX DMARDs or biologic monotherapy).

## 4. Discussion

Methotrexate (MTX), one of the most widely used disease-modifying antirheumatic drugs (DMARDs) in the treatment of rheumatoid arthritis (RA), has significantly improved the course of the disease, enhanced patients’ quality of life, and reduced disease-related mortality [6,7]. Currently, MTX is considered the most effective therapeutic agent in preventing radiological progression; however, in cases of MTX treatment failure, combination therapy with additional DMARDs or biologic agents is preferred [7]. Pharmacologically, MTX exerts antifolate and antimetabolite effects by inhibiting dihydrofolate reductase, thereby reducing purine and pyrimidine synthesis. It possesses numerous biochemical effects, including impacts on DNA and RNA synthesis, T-cell apoptosis, cell proliferation, and cytokine production [4,8]. Depletion of hepatic folate stores and local folate deficiency-related toxicity is among the possible hepatotoxic effects of MTX. Nevertheless, a definitive experimental correlation between folate depletion and hepatotoxicity has not been established [9]. Thus, while the efficacy of MTX is widely accepted, hepatotoxicity remains one of the major limiting factors in treatment [10]. Supplementation with folic acid has been shown to reduce the frequency and severity of hepatotoxicity without diminishing its anti-inflammatory therapeutic effects [11,12]. Sotoudehmanesh R et al. reported an incidence of hepatotoxicity exceeding 20%, emphasizing its association with cumulative dose [13]. Another important cause of MTX-related adverse effects is the use of daily or high-dose therapeutic regimens in earlier applications, where significant toxicity was observed [3]. A spectrum of hepatic adverse effects, ranging from asymptomatic transaminase elevations to fibrosis and even fatal hepatic necrosis, has been associated with dose dependency [4,14]. Concerns regarding potential liver toxicity may lead to avoidance or discontinuation of treatment, or to the initiation of costly, invasive, and potentially unnecessary investigations. Moreover, extrapolating adverse effects observed with high-dose MTX in oncological settings to modern low-dose MTX regimens used in autoimmune diseases may lead to misinterpretations during treatment monitoring [3]. Following high-dose intravenous MTX, transaminases have been reported to rise up to 10–20 times the upper limit of normal (ULN), but they typically normalize rapidly without causing jaundice or permanent liver damage in most cases [1]. In contrast, in low- to moderate-dose regimens, ALT/AST elevations are generally mild and improve with dose reduction or treatment discontinuation [4]. With current monitoring strategies and novel therapeutic regimens, risks historically associated with MTX use appear to have decreased substantially. Two recent high-quality studies reported transaminase elevations in 22% of patients, but only 1% exceeded twice the ULN [15,16]. Nonetheless, higher liver enzyme levels are observed when MTX is used in combination regimens [2,7,17]. A recent systematic review of 18 studies, comprising 2199 patients, evaluated MTX-related hepatotoxicity at an average weekly dose of 12.5 mg. The cumulative incidence of elevated liver transaminases was 49%. However, only 24% of patients received folic acid supplementation, while 56% were concurrently treated with nonsteroidal anti-inflammatory drugs. As a consequence of elevated liver transaminases, MTX dose was reduced in 26% of patients and permanently discontinued in 7% [5]. In our study, none of the patients received high-dose MTX; instead, a daily low dose of 7.5 mg was administered, and all patients were supplemented with folic acid. This may have contributed to the mild nature of the observed transaminase elevations. Among our patients, dose reduction was sufficient in five cases with enzyme elevations exceeding three times the ULN, while MTX discontinuation was required in two patients. As all of these patients were on combination therapy, it suggests that MTX alone may not have been the sole causative agent.

In clinical guidelines, regular monitoring of liver enzymes at 8–12 week intervals is recommended in patients treated with methotrexate (MTX) [5,18]. Similarly, in our study, liver transaminases were monitored at 8–12-week intervals following early assessments at weeks 4–6. The study by Kremer et al. formed the basis of the 1994 American College of Rheumatology (ACR) guidelines, recommending AST/ALT monitoring every 4–8 weeks, and proposing liver biopsy (LB) if abnormalities were observed in five out of nine tests during the first year of therapy [19]. Baseline transaminase levels prior to MTX initiation are also of importance; stable enzyme levels during therapy do not necessitate further intervention. In a meta-analysis by Visser et al., pooled data from 12 out of 18 studies demonstrated a cumulative incidence of elevated liver enzymes of 31%. When classified by AST/ALT thresholds, the cumulative incidence was 49% above the upper limit of normal (ULN) and 17% above 2–3 × ULN. Among patients with elevated liver enzymes, MTX therapy was continued without dose adjustment in 67%, while dose reduction or temporary discontinuation was implemented in 26%, and permanent discontinuation occurred in 7% [5]. Guidelines, including the widely used ACR recommendations, have also reported that elevated baseline ALT levels prior to MTX initiation serve as predictors of ALT elevations during treatment [16]. Ujfalussy et al. and Weinblatt et al. reported that after 5–6 years of follow-up in RA patients, 5% and 2%, respectively, discontinued MTX permanently due to recurrent liver enzyme elevations [20,21]. In a study by Johanna Karlsson Sundbaum et al., 213 RA patients initiating MTX were followed for a mean of 4.3 years, with a total of 6288 ALT tests performed. During follow-up, ALT > 1.5 × ULN was observed in 44 patients (21%), with elevated baseline ALT identified as the strongest predictor. Among patients who continued therapy, 70% experienced recurrent elevations, and outcomes were similar between those who underwent dose adjustments and those who did not (67% vs. 73%, *p* = 0.43). Seven patients (3%) permanently discontinued MTX due to ALT elevations, but no cases of liver failure were reported [22]. In our study, over an average follow-up period of 3 years, dose reduction was required in 5 patients, while treatment was permanently discontinued in 2 patients due to markedly elevated liver enzymes. Notably, both patients who discontinued MTX had elevated baseline liver enzyme levels prior to treatment, supporting the literature that emphasizes pretreatment liver enzyme levels as an important predictor of subsequent hepatotoxicity. In the study by Anvari et al., it was emphasized that normalization rates of liver enzymes were high following MTX dose reduction or discontinuation (e.g., 93% or 65%), whereas recovery rates were lower when the dose was maintained [23]. After a comprehensive evaluation, if no alternative cause is identified beyond MTX, guideline-recommended therapeutic approaches depend on the degree of transaminase elevation. Although the threshold for immediate MTX withdrawal varies across guidelines, elevations exceeding three times the upper limit of normal (ULN) are generally considered significant. Persistent low-grade elevations, particularly when progressive trends are observed, may also necessitate intervention [24]. In our study, we similarly applied the threshold of 3 × ULN, implementing dose reduction in 5 cases and discontinuation in 2 cases in accordance with the literature. Kremer et al. prospectively followed RA patients on long-term MTX therapy for a mean of 7.5 years, reporting that enzyme elevations were most frequently observed during the initial years of treatment [25]. Rau et al. likewise found that liver enzyme elevations occurred most commonly in the early months of therapy [26]. In our study, early monitoring of the 15 patients with baseline elevations revealed that 86% exhibited abnormalities in the first years of treatment. A substantial proportion of these patients (*n* = 26) subsequently demonstrated normalization during therapy, consistent with the predictive value of early ALT elevations reported in the literature [22,23]. In a study evaluating histological findings, Quintin et al. suggested that with low-dose, long-term MTX therapy, direct hepatotoxic effects are limited, and enzyme elevations may be more strongly associated with underlying metabolic or autoimmune conditions [27]. Evidence regarding the correlation between enzyme elevations and histological abnormalities is inconsistent. Some studies reported an association between recurrent abnormal enzyme measurements and histological abnormalities [28,29]. Walker et al., in case–control analyses, showed that RA patients treated with MTX who had clinically significant liver disease confirmed by biopsy exhibited higher rates of enzyme elevations compared to those without liver disease (29). One study assessing liver biopsy (LB) results before and after MTX therapy reported mild fibrosis, severe fibrosis, and cirrhosis rates of 9.1%, 0%, and 0.3% at baseline, compared with 15.3%, 1.3%, and 0.5% after MTX therapy, respectively [5]. Among four studies investigating predictors of liver fibrosis/cirrhosis through extensive statistical analyses, two identified cumulative MTX dose and treatment duration as significant determinants [28,29]. A literature review of biopsy-proven hepatic abnormalities reported that 3% of patients developed histological abnormalities after one year of MTX therapy; however, importantly, no histological progression was observed after four years of treatment [30]. In our study, the absence of baseline and post-treatment biopsy results represents a limitation; nonetheless, the lack of indication for biopsy in any case supports the absence of histological worsening under MTX therapy.

Recent studies indicate that MTX carries a side-effect and toxicity profile comparable to other agents, although combination regimens may confer a higher risk of infections and hepatic adverse events [7]. In studies by Gibofsky et al., combination regimens, particularly MTX + leflunomide, were associated with increased hepatotoxicity risk, with ALT >3 × ULN occurring in 3.8% of combination users versus 0.8% of MTX monotherapy users [12]. Similarly, the literature highlights that leflunomide + MTX combination therapy is particularly associated with higher hepatotoxicity risk [12,15]. In a large cohort study using CORRONA registry data, Curtis JR reported ALT/AST elevations in 14–22% of patients on MTX or leflunomide monotherapy, compared with 31% in the MTX + leflunomide group, further supporting the increased hepatotoxicity risk of combination regimens [15]. Large epidemiological studies have also demonstrated that biologic DMARDs carry a higher risk of both serious and non-serious hepatic adverse events compared with MTX or leflunomide (e.g., RR = 5.5; 95% CI 1.2–24.6 for serious events) [31]. Use of MTX in combination with biologic agents can also result in increased liver enzyme levels. Rigby WFC et al. reported ALT/AST elevations of 4–6% with golimumab + MTX compared with lower rates in monotherapy. Similarly, ALT > 3 × ULN was observed in 7.7% of patients on tocilizumab + MTX, compared with only 1.2% on tocilizumab monotherapy [32]. Kremer JM et al. and Singh JA et al. likewise reported higher rates of liver enzyme elevations with biologic therapies, particularly when used in combination with MTX [33,34]. This finding suggests that the effect may not stem from direct hepatotoxicity of biologics, but rather from the added burden of immunomodulation and polypharmacy on hepatic metabolism. In our findings, patients on MTX monotherapy generally exhibited mild enzyme elevations (<2 × ULN) (*n* = 11, Table 2), whereas more severe elevations were observed in those receiving MTX in combination with DMARDs or biologics (*n* = 15, Table 2). Among patients on combination therapy, 7 required MTX dose reduction, while in 2 cases significant elevations necessitated discontinuation, with subsequent improvement. In conclusion, liver enzyme elevations in RA patients under MTX monotherapy are generally mild and transient; however, more pronounced abnormalities may occur with combination therapy. Careful assessment of baseline enzyme levels and close laboratory monitoring during treatment are important, and timely intervention strategies such as dose reduction or drug discontinuation can effectively manage the risk of hepatotoxicity. Strengths of our study include the exclusion of potential hepatic risk factors such as alcohol use, chronic hepatitis, and NAFLD, as well as the provision of folic acid supplementation to all patients. Nevertheless, the retrospective design and the lack of advanced assessments such as liver biopsy represent limitations. Larger, prospective, biopsy-based studies are warranted to more clearly elucidate these associations.

## Figures and Tables

**Table 1:** Demographic and Clinical Characteristics.

Characteristics	All Patients (*n* = 33)	Female (*n* = 19)	Male (*n* = 14)
Age (years, mean ± SD)	66 ± 11	68 ± 10	64 ± 12
Comorbidity (present, %)	12 (36%)	7 (37%)	5 (36%)
Polytherapy (%)	15 (45%)	9 (47%)	6 (43%)
MTX monotherapy	12 (36%)	7 (37%)	5 (36%)
Non-MTX monotherapy	4 (12%)	2 (11%)	2 (14%)
MTX + other DMARD	8 (24%)	5 (26%)	3 (21%)
Biologic monotherapy	2 (6%)	1 (5%)	1 (7%)
MTX + Biologic agent	7(21%)	4(23%)	3(22%)

**Table 2:** Distribution of liver enzyme elevations by degree.

Degree of Enzyme Elevation	Number of Patients	Percentage (%)	Female/Male
1–2 × ULN	22	67%	6/4
2–3 × ULN	7	21%	5/3
>3 × ULN	4	12%	8/7
Total	33	100%	19/14

**Table 3:** Distribution of Patients by Treatment Type and Degree of Liver Enzyme Elevation.

Treatment Type	All Patients (*n* = 33)	1–2 × ULN	2–3 × ULN	>3 × ULN
MTX monotherapy	12	11	1	0
Non-MTX monotherapy	4	4	-	-
MTX + other DMARD	8	5	2	1
Biologic monotherapy	2	1	1	0
MTX + Biologic agent	7	1	3	3

**Table 4:** Distribution of Patients with Elevated Liver Enzymes Before Treatment.

Degree of Enzyme Elevation	All Patients (*n* = 15)	MTX Monotherapy	Non-MTX Monotherapy	Polytherapy (%)
1–2 × ULN	8 (53%)	5	2	1
2–3 × ULN	6 (40%)	0	0	6
>3 × ULN	1 (7%)	0	0	1

## Data Availability

The data supporting the findings of this study are available from the resources of İnönü University Faculty of Medicine Hospital.
